# Cognitive Behavioural Therapy and Light Dark Therapy for Maternal Postpartum Insomnia Symptoms: Protocol of a Parallel-Group Randomised Controlled Efficacy Trial

**DOI:** 10.3389/fgwh.2020.591677

**Published:** 2021-01-15

**Authors:** Sumedha Verma, Shantha M. W. Rajaratnam, Margot Davey, Joshua F. Wiley, Bei Bei

**Affiliations:** ^1^Faculty of Medicine, Nursing and Health Sciences, School of Psychological Sciences, The Turner Institute for Brain and Mental Health, Monash University, Clayton, VIC, Australia; ^2^Melbourne Children's Sleep Unit, Monash Medical Centre, Clayton South, VIC, Australia; ^3^Department of Psychiatry, Centre for Women's Mental Health, Royal Women's Hospital, University of Melbourne, Parkville, VIC, Australia

**Keywords:** postpartum, sleep, insomnia, bright light therapy (BLT), cognitive behavioural therapy (CBT), circadian rhythm, randomised controlled trial

## Abstract

**Background:** Symptoms of insomnia are common in new mothers and have been associated with a range of negative maternal and child outcomes. Despite this, interventions to improve maternal postpartum sleep remain scarce. Cognitive Behavioural Therapy (CBT) and Light Dark Therapy (LDT) represent two promising interventions for insomnia symptoms and associated daytime consequences such as fatigue. This randomised controlled trial examines whether CBT and LDT improve maternal insomnia symptoms as the primary outcome and maternal sleep disturbance, mood, fatigue, and sleepiness as secondary outcomes. This protocol paper outlines the development, design, and implementation of the trial.

**Methods:** Participants are an Australian community-sample of 90 first-time mothers who are 4–12 months postpartum with self-reported symptoms of insomnia (Insomnia Severity Index scores ≥ 8). Exclusion criteria include current severe sleep/psychiatric disorders, unsettled infant sleep behaviour, sleep-affecting medication use, and photosensitivity. Eligible women are randomised into a CBT (strategies targeting sleep, worries, fatigue, and relaxation), LDT, or a treatment-as-usual control condition. Interventions are therapist-assisted and personalised through two telephone calls and include a series of automated intervention emails delivered over 6 weeks. Primary and secondary outcomes are assessed at four time points: baseline, intervention mid-point, post-intervention, and 1-month post-intervention.

**Discussion:** If found effective, these interventions could represent efficacious, safe, and inexpensive treatments for improving postpartum insomnia and mitigate its negative impact on maternal well-being. Interventions tested are highly scalable and can be integrated into postpartum care and made available to the broader community.

**ANZCTR trial registration:** Accessible at: https://anzctr.org.au/Trial/Registration/TrialReview.aspx?ACTRN=12618000842268.

## Introduction

Following childbirth, women experience inconsistent sleep-wake times, overnight awakenings and daytime napping to compensate for poor nighttime sleep (1–6). While some aspects of sleep (e.g., sleep duration) may eventually improve over time, others (e.g., sleep efficiency) continue to be compromised beyond the initial postpartum months (2, 3) with a significant proportion (41%) of women reporting significant insomnia symptoms at 2 years postpartum (7).

Maternal insomnia symptoms have been associated with a range of negative outcomes including mood disturbance (8–11), fatigue (12, 13), and family dysfunction (14). While there has been some progress in infant sleep interventions (15, 16), evidence-based interventions to aid maternal sleep during the postpartum period, despite being imperative, remain notably limited (17).

Postpartum insomnia symptoms may develop via different mechanisms. From cognitive behavioural perspectives, disturbed sleep experienced during pregnancy and early postpartum may precipitate unhelpful sleep behaviours (e.g., spending excessive time in bed) and cognitions (e.g., worries about consequence of poor sleep), which may perpetuate symptoms of insomnia. Cognitive Behavioural Therapy (CBT) for insomnia is a non-pharmacological intervention that targets underlying cognitive and behavioural factors that perpetuate insomnia through various recommendations such as stimulus control, sleep restriction, cognitive strategies, relaxation training, and sleep hygiene (18); it is shown to be a safe treatment option (19) and highly effective for reducing insomnia in diverse populations (20, 21), including in pregnant women with insomnia (22, 23). Even while external factors (e.g., infant awakenings) disrupt sleep, CBT remains effective in that it increases sleep drive, reduces conditioned arousal and alters maladaptive cognitions and behaviours underlying insomnia (18). A recent randomised controlled trial (RCT) of CBT for insomnia during pregnancy demonstrated significant improvements in insomnia symptoms for women in the intervention condition compared to those in the control (22).

A second potential mechanism of postpartum insomnia is circadian rhythm disturbance. Dampened circadian amplitude and phase delays have been reported during perinatal periods (11, 24, 25). It is possible that altered sleep-wake timing (3, 26) and low levels of light exposure (27) during postpartum periods may underlie these changes. Circadian disruption has been linked to symptoms of insomnia (28) and mood disturbance (29–32). Light therapy involves appropriately-timed light exposure to realign the circadian clock with externally required sleep/wake timing (33) and/or to reduce sleepiness through its direct alerting effects (34). There may also be provision of strategies to reduce light exposure at night (i.e., Dark Therapy), such as through the use of light-blocking glasses (33, 35). Light therapy has been shown to be safe (33) and an effective intervention for improving sleep disturbance (36), fatigue (37), and mood (38), including depression in both general (39) and perinatal (40) populations. The benefits of light therapy for improving postpartum insomnia symptoms and associated daytime consequences such as postpartum fatigue, however, remains to be elucidated.

The aim of the current study is to simultaneously evaluate the efficacy of CBT and Light Dark Therapy (LDT), two distinctive interventions targeting two different potential mechanisms (i.e., cognitive-behavioural and circadian) of postpartum insomnia to accelerate intervention development. It is hypothesised that compared to a treatment-as-usual (TAU) control condition, women who receive either intervention will demonstrate significantly greater improvements in maternal insomnia symptoms as the primary outcome, and maternal sleep disturbance, fatigue, sleepiness, and symptoms of depression and anxiety as secondary outcomes. This comparator was selected as an ideal control to assess efficacy of interventions compared to existing clinical practices of routine perinatal care (41).

## Methods

### Design

**Figure 1:**
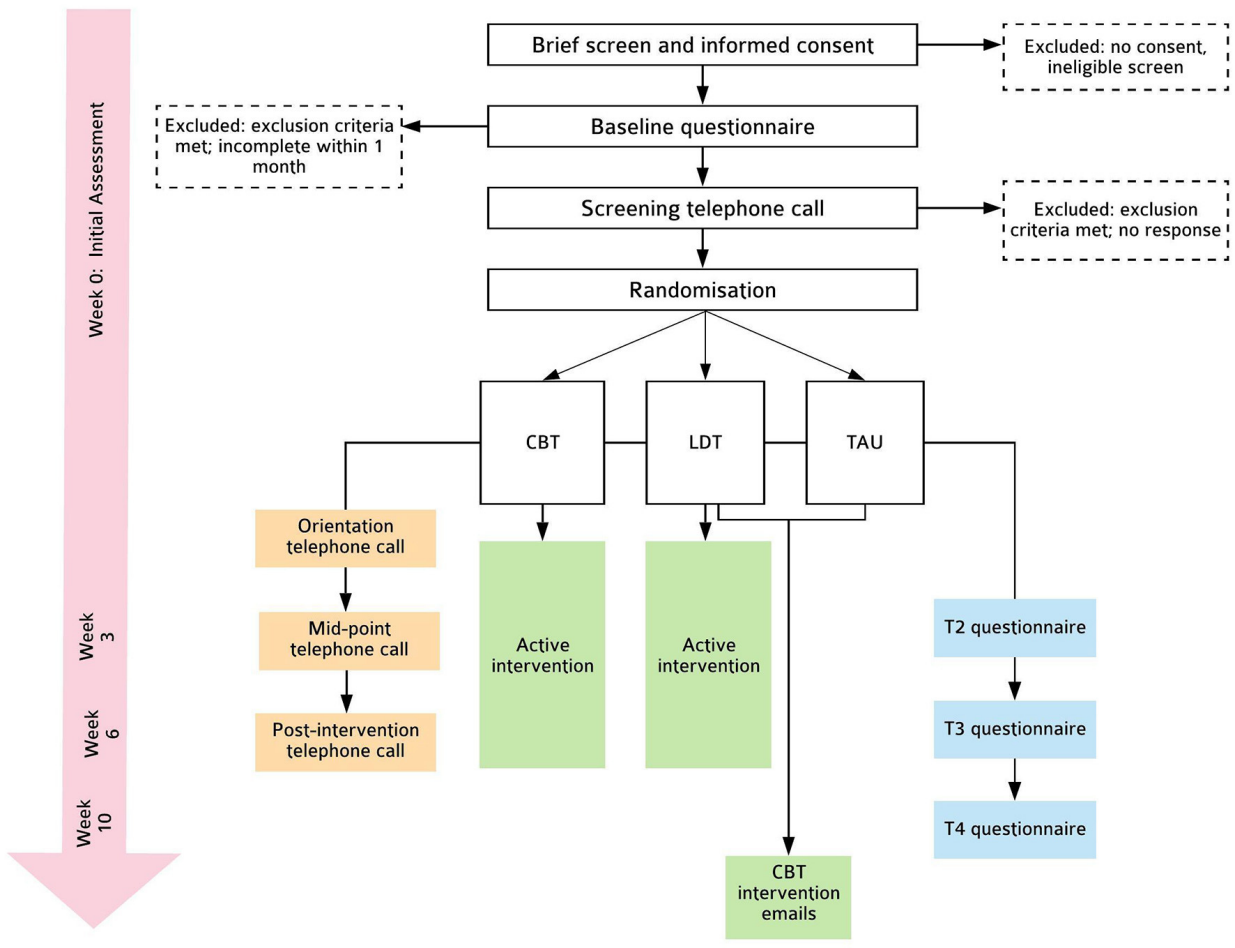
Flowchart of trial procedures.

This is a parallel-group, randomised controlled efficacy trial, consisting of two intervention conditions (CBT and LDT) and one TAU control condition, randomised in a 1:1:1 ratio. Outcomes are assessed at baseline (Week 0; T0), intervention mid-point (Week 3; T2), post-intervention (Week 6; T3), and 1-month follow-up (Week 10; T4). This protocol follows SPIRIT-PRO (42) and TIDieR (43) guidelines (see Supplementary Material). Please see Figure 1 for details.

### Participants

Inclusion criteria are: (a) nulliparous women (i.e., first-time mothers with no older children) 4–12 months postpartum; (b) age ≥18 years; (c) reports symptoms of insomnia, operationalised as scoring ≥8 (subthreshold insomnia) on the Insomnia Severity Index [ISI; (44)]; (d) singleton pregnancy; (e) ability to read and write in English; (f) regular access to telephone, email and the internet. Nulliparous women were chosen given the higher risk of poorer sleep compared to multipara (2, 45, 46).

Exclusion criteria are: (a) current pregnancy; (b) night shift work; (c) current severe or untreated sleep disorders (e.g., narcolepsy, sleep apnea, restless legs syndrome, circadian rhythm disorders); (d) take medications or have medical conditions that directly affect sleep (e.g., sleep medication, antidepressants); (e) report current severe psychopathology (e.g., bipolar disorder, post-traumatic stress disorder) or are at high risk of harm to self/others; (f) have infants with medical conditions that affect sleep; (g) suffer from epilepsy; (h) report photosensitivity or have been recommended by health professional to avoid bright light; (i) report current unsettled infant sleep behaviour, operationalised as infant waking on average >3 times per night and requiring parental assistance to reinitiate sleep; this final exclusion criterion was established as this trial focuses on the management of maternal insomnia symptoms rather than sleep deprivation primarily due to infant care. Participants are permitted to receive ongoing usual care for physical and/or mental health conditions.

### Procedure

#### Recruitment

The study is referred to as the Postpartum Sleep Study for Mothers and a mixture of community and online recruitment are used. Facebook groups for parents within Australia are approached to post a “call for participants” containing information about the trial alongside a link to the online explanatory statement. Various maternal and child health centres within metropolitan Melbourne are contacted to assist with distribution of project information. Relevant Australian organisations and media outlets are contacted with details of the project and asked encouraged to share information with consumers.

#### Informed Consent

The explanatory statement includes comprehensive details of the project such as information regarding interventions, assessments, research procedure, potential risks, reimbursement, confidentiality, and freedom to withdraw participation. After reading the online explanatory statement, participants proceed to complete brief screening questions before providing informed consent (see Supplementary Material). Women <4 months postpartum may still provide their consent and are contacted once they have reached the appropriate time frame for inclusion. Women who do not fulfil initial eligibility are directed to a list of further support services.

#### Telephone Interview and Screening

Baseline questionnaire responses are reviewed and respondents who do not yet met exclusion criteria are contacted to undertake a telephone screening interview to assess further eligibility. The telephone interview covers: the Duke Structured Interview for Sleep Disorders [DSISD; (47)], sleep/wake habits, medication use, engagement in psychotherapy or sleep treatments, and a risk assessment. Women ineligible upon initial screening are provided with a list of suitable support services, discussed with them via telephone and reiterated in a follow-up email.

##### Risk Assessment

A risk assessment is undertaken during the initial telephone interview, as well as during mid-point and post-intervention telephone calls (discussed further). It assesses suicidal/homicidal ideation, plan, history of injurious behaviour, and engagement with mental health care. During the initial interview, women deemed to be at high risk, operationalised as current suicidal/homicidal ideation with plan and/or history of harm, are excluded from the study and referred to relevant services for clinical care. Women at medium risk, operationalised as experiencing current depression with ideation to harm self/others but have no plan, are included if no exclusion criteria have been met; these women are encouraged to seek professional help for their mental health via general practitioner or psychologist, and will also be provided with details of relevant mental health services. Women at low risk are included if no exclusion criteria are met.

Initial and mid-point telephone calls are carried out by a provisional psychologist undertaking doctoral training in clinical psychology (SV) who consults a clinical psychologist (BB) when any risk issues arise to decide on appropriate action. Risk assessments in the post-intervention telephone call are undertaken by research assistants who received training by a provisional (SV) and clinical psychologist (BB). Research assistants contact the provisional psychologist (SV) if any risk issues arise, who consults the clinical psychologist (BB) to decide on appropriate action.

#### Randomisation and Blinding

Eligible participants are randomised using a complete randomisation scheme generated in advance. Specifically, block sizes of variable size (3, 6, 9) are used. Random seeds are generated to assure allocation concealment and pre-guessing of the allocation sequence at the end of each block. Randomisation are stratified by baseline ISI (≤ 14 and >14) and infant age (<8 months and ≥8 months). As noted on trial registration, this protocol commenced as a four-arm trial with the fourth arm receiving both CBT and LDT. Due to significant and unexpected changes in the first author's personal circumstances during the early phase of this trial, this fourth arm was removed after 31 participants were randomised (9 to the removed condition) to ensure timely trial completion. Randomisation used during this initial phase was identical to subsequent 3-arm randomisation described above, except the randomisation scheme had block sizes of 4 or 8.

The randomisation scheme is generated in R (48) and is set up in REDCap by a member of the research staff who is not involved in recruitment or delivery of intervention and not a principal investigators. To randomise a participant, an authorised research staff member logs into REDCap, enters eligibility and participant stratification data before receiving group allocation. Participants are not blinded but are asked to withhold group allocation from research assistants during the post-intervention telephone call.

#### Procedures

Eligible participants are invited to complete questionnaires on Qualtrics software (49) and receive automatic prompts to ensure all items are responded to, and those who do not complete questionnaires within 1 week are reminded. Once randomised, participants receive interventions as outlined below. A final telephone call (5–10 min) by a research assistant blinded to group allocation is made to all participants. In this telephone call, the DSISD Insomnia module is administered to determine the presence/absence of insomnia disorder and assess risk. All telephone communications are audio-recorded for assessing reliability and intervention fidelity.

### Interventions

All participants, regardless of group allocation, continue usual postpartum care. No participants are restrained from seeking treatment or support for sleep concerns. Treatment or support received outside of the study interventions are documented.

Interventions include therapist-assisted and self-directed/automated components which are delivered via distance. Interventions are manualised and telephone scripts were developed by the research team to ensure consistency. A provisional psychologist undertaking doctoral training in clinical psychology (SV) who was trained prior to trial commencement in CBT for insomnia delivery by a clinical psychologist (BB) carries out intervention telephone calls and receives regular supervision throughout the trial.

#### Cognitive Behavioural Therapy

The CBT material is based on CBT for insomnia adapted for perinatal populations (22, 50). This mode of treatment has been identified as the intervention of choice by pregnant women and their partners compared to pharmacotherapy and no treatment (51). To adapt intervention for later postpartum months, a focus group with four new mothers was carried out to aid intervention development and provided guidance on content and delivery format. Strategies are delivered via emails which are designed to be visually-attractive, easy-to-read and brief (see Supplementary Material), in light of recommendations provided in the focus group. Components of the CBT intervention include: psychoeducation, stimulus control, healthy attitudes and behaviours around sleep, sleep hygiene, relaxation, managing unhelpful thoughts and worries, fatigue management, prioritising rest and self-care, enlisting social support and infant settling. Emails are delivered using an online software that provides professional email templates and automates the timing of intervention delivery based on each woman's own progress. In total, 21 emails are delivered: five in Week 1, four in Week 2, followed by three emails per week in Weeks 3 to Week 6.

#### Light Dark Therapy

Participants in the LDT group are provided with light therapy glasses (Luminette 2) and are asked to wear them for at least 20 (but no more than 30) min each morning immediately upon waking. The light glasses are fixed at the brightest factory setting and provide white light [Correlated Colour Temperature (CCT) = 5,260 Kelvin] which is blue-enriched with a peak spectral wavelength of 466 nm at an intensity of 1,250 lux directed at each eye measured via spectrophotometry. The melanopic lux of the light glasses was 813 lux, calculated using the International Commission on Illumination system [CIE; (52)]. Light glasses have received the EC Full Quality Assurance Certificate from SGS, Belgium (Directive - 93/42/EEC). Participants receive a Luminette User Manual for further detailed information regarding their light glasses. Participants are also provided light hygiene strategies regarding strategies regarding light exposure during both day and night. These include seeking natural bright light to promote alertness during the day and light avoidance in the hours prior to nighttime sleep, especially blue light from hand-held electronic devices. A small LED night light (Dreambaby Auto Sensor Swivel Light) is provided to participants to use during nighttime awakenings (e.g., overnight infant care). The night light has been tested using spectrophotometry and yields 5 lux at 12 inches distance (CCT = 5,754 Kelvin) and 4 melanopic lux (52). Brief weekly emails are sent from Week 1 to Week 6 to remind participants of pertinent strategies and encourage intervention adherence. After completing final assessments at T4, LDT participants receive the CBT intervention emails in bulk.

#### TAU Control

Participants in the TAU condition complete assessments at all time points and continue to receive usual postnatal care. After completing final assessments at T4, TAU participants receive CBT intervention emails.

#### Therapist Assistance

Participants in CBT and LDT conditions receive two scheduled telephone calls with a provisional psychologist: (1) an orientation telephone call in Week 1 which aims to: (a) explain key components of the interventions, (b) provide personalised recommendations, and (c) encourage intervention adherence, and (2) a mid-point telephone call. Participants in intervention conditions all receive core components of interventions as listed above however components specifically relevant to the individual are highlighted. For example, relaxation strategies may be highlighted for a participant who reports feelings of tension around sleep onset or addressing dysfunctional beliefs about sleep for another participant who presents with high sleep-related anxiety. LDT is personalised by providing a specific time to begin light glasses usage based on habitual wake time and selecting an optimal location for the night light in the home.

Orientation telephone calls takes ~45 min for those in the LDT condition and 60 min for those in the CBT condition. Following the orientation telephone call, LDT participants are provided with a two-page summary of key points, including reminder of personalised time to commence light glasses usage in the morning. During the orientation telephone call, TAU participants are thanked for their involvement and reminded of project logistics (~10 min).

##### Mid-point Telephone Call

All participants receive a telephone call in Week 3 (for CBT and LDT group, this is intervention mid-point). The purpose of the telephone call for those in intervention conditions is to ascertain and encourage intervention adherence, and problem-solve any obstacles to utilising intervention strategies. Participants in intervention conditions are encouraged to keep using intervention strategies beyond the sixth week. Those in the TAU condition are thanked for their ongoing participation and discuss any questions related to logistic aspects of participation. As previously mentioned, a risk assessment is undertaken in the mid-point telephone call.

### Adverse Events

Risk is minimised by early exclusion of women for whom participation may pose a risk of harm (e.g., those with photosensitivity, severe psychopathology, epilepsy etc.). Any risk issues are documented in a tracking sheet, including any internal consultations and actions decided (e.g., referral following exclusion) by the research team. During screening, a physician with extensive experience in sleep medicine is consulted when clarification of inclusion/exclusion criteria is required (e.g., use of medication, medical conditions).

Side-effects of participation are assessed in the two final questionnaires. Participants are asked to report any side-effects of LDT immediately so that intervention may be appropriately adapted (e.g., ensuring sufficient ambient light exposure during glasses usage, delaying use of glasses, substitution of glasses usage with natural light exposure). Women who report side-effects are monitored via telephone and email to ensure adherence with recommendations to minimise harm. A physician is consulted in the event of adverse events. Any adverse events that occur throughout the trial are recorded in a tracking sheet.

Any protocol modifications are first approved by Monash University Human Research Ethics Committee before being implemented and communicated to participants if relevant. Long-lasting, serious harm is not expected as a result of trial participation which is explicated in the explanatory statement.

### Measurements

Questionnaires include various self-report measures of sleep, mental health, fatigue, perceived stress, social support, self-efficacy, and relationship satisfaction among other factors. Self-report sleep measures with strong psychometric properties were selected for primary and secondary outcomes as self-reported sleep complaints (not objectively-assessed sleep parameters) share strong associations with other well-being outcomes (53). Further, insomnia, the primary outcome of this trial, is assessed and diagnosed based on self-reported sleep complaints (54). To reduce study burden for this vulnerable population, we did not include objective measures of sleep. Please see Table 1 for a list of measures and their associated timing.

**Table 1:** Timing of measurements.

	**Enrolment**	**Allocation**	**Post-allocation**			
**Timepoint**	**T0**	**0**	**T1**	**T2**	**T3**	**T4**
**Enrolment**						
Consent and Screen	X					
Randomisation		X				
**Intervention**						
CBT			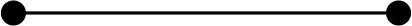			
LDT			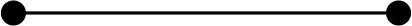			
TAU			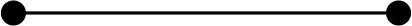			
**Structured interviews**						
Duke structured interview for sleep disorders	X				^*^	
**Primary outcome**						
Insomnia severity index	X			X	X	X
**Secondary outcomes**						
PROMIS sleep disturbance – SF	X			X	X	X
Fatigue assessment scale (5-item, modified)	X			X	X	X
Visual analog scale of fatigue	X			X	X	X
PROMIS anxiety – SF	X			X	X	X
PROMIS depression – SF	X			X	X	X
Epworth sleepiness scale	X			X	X	X
Karolinska sleepiness scale	X			X	X	X
**Other factors**						
Demographic and obstetric information	X				^∧^	^∧^
Postnatal questions on sleep timing, duration and quality	X			X	X	X
Dysfunctional beliefs about sleep scale	X			X	X	X
Morningness-eveningness questionnaire	X			X	X	X
Glasgow sleep effort scale	X			X	X	X
Brief infant sleep questionnaire	X			X	X	X
Perceived stress scale	X			X	X	X
PROMIS emotional support – SF	X			X	X	X
PROMIS instrumental support – SF	X			X	X	X
Maternal efficacy questionnaire	X				X	X
Mother to infant bonding scale	X				X	X
Dyadic adjustment scale – 4	X				X	X
Cross-cutting symptoms level 1	X				X	
Ford insomnia response to stress test	X					
Maternal sleep history questions	X					
Credibility expectancy questionnaire	X					
Intervention adherence and usefulness^#^					X	^∧^
Client satisfaction questionnaire^#^					X	
COVID-19 questions						X

*T0, Pre-study screening/consent and baseline; T1, Time-point 1 (Week 0) intervention start; T2, time-point 2 (Week 3) intervention mid-point; T3, time-point 3 (Week 6) immediate post-intervention; T4, time-point 4 (Week 10) 1-month follow-up; CBT, Cognitive Behavioural Therapy; LDT, Light Dark Therapy; TAU, treatment-as-usual; X, Measure administered at that time point*;

* *Insomnia module administered only (10 mins)*;

∧ *only a subset of questions asked*;

# *not administered to TAU; SF, short form*.

#### Primary Outcome

The ISI is a 7-item measure of self-reported insomnia symptoms (44) and is used as the primary outcome. Total scores range from 0 to 28, with higher scores indicating more severe insomnia symptoms. Items such as “*difficulty falling asleep*” and “*difficulty staying asleep*” are rated on a 5-point Likert-type severity scale (“*during the past 2 weeks*”), ranging from 0 = none to 4 = very severe. Scores of 0-7 = no clinical insomnia symptoms; 8–14 = sub-threshold insomnia; 15–21 = moderate clinical insomnia; and 22–28 = severe clinical insomnia (55). The ISI has been shown to have high internal consistency and a cutoff score of ≥8 has a sensitivity 96–99% and specificity of 78–92% in community and clinical populations respectively (56).

#### Secondary Outcomes

1. Maternal sleep quality, measured using PROMIS Sleep Disturbance – Short Form – 8a (57), a brief, 8-item instrument assessing sleep disturbance.
2. Fatigue, measured using a 5-item adapted version of the Fatigue Assessment Scale for use in the postpartum population (58) and the Visual Analog of Fatigue Scale (59).
3. Maternal mood, measured with PROMIS Depression – Short Form – 8a and PROMIS Anxiety – Short Form – 8a (60) which measure symptoms of depression and anxiety respectively.
4. Trait and state sleepiness, measured using the Epworth Sleepiness Scale (61) and Karolinska Sleepiness Scale (62) respectively.

#### Other Factors

1. Demographic, medical conditions, and obstetric information are collected using self-report at baseline (T1) and changes are monitored at T3 and T4 (e.g., infant-feeding method, medical conditions of mother/infant, engagement in therapy, medication use).
2. Postnatal Sleep Questions include self-reported maternal sleep behaviours over the past week (e.g., sleep duration, onset latency, wake after sleep onset, daytime naps adapted from the Consensus Sleep Diary (63).
3. Self-reported maternal sleep history (i.e., pre-pregnancy sleep) are assessed using adapted versions of the PROMIS-SD-SF and Postnatal Sleep Questions.
4. Maternal mental health, measured with the Cross-Cutting Symptoms Level 1 (64).
5. Mother-infant attachment quality, assessed using the Mother to Infant Bonding Scale (65).
6. Relationship satisfaction with the partner, measured using the brief Dyadic Adjustment Scale – 4 (66).
7. The following are assessed and examined as potential covariates: infant sleep [Brief Infant Sleep Questionnaire; (67)]; perceived stress [Perceived Stress Scale; (68)]; social support measured using the PROMIS Instrumental Support – Short Form – 4a and PROMIS Emotional Support – Short Form – 4a (69); questions on the partner's sleep behaviour and overnight assistance with infant care are also included.
8. Feasibility and acceptability are assessed implicitly via recruitment and dropout rates, and explicitly via the Client Satisfaction Questionnaire (70) and qualitative feedback from participants.
9. Due to ongoing data collection during the COVID-19 pandemic, questions related to personal experiences with COVID-19 and potential impact on participation are asked to all participants who completed the project after mid-February 2020 (shortly before the outbreak was declared a national pandemic in Australia). This is intended to assist with interpreting findings from this trial following completion.

The following constructs will also be measured and explored as predictors or mediators of treatment responses:

1. Vulnerability to insomnia under stress, using the Ford Insomnia Response to Stress Test (71).
2. Maternal self-efficacy, assessed using the Maternal Efficacy Questionnaire (72).
3. Beliefs and attitudes about sleep, using the Dysfunctional Beliefs and Attitudes about Sleep Scale (73).
4. Chronotype via the Morningness Eveningness Questionnaire (74).
5. Sleep effort, using the Glasgow Sleep Effort Scale (75).
6. Participants' perceived credibility and expectancy of treatment via the Credibility Expectancy Questionnaire (76).
7. Intervention adherence (assessing frequency and usefulness of each intervention component).

### Reimbursement

Upon completion of the final questionnaire and return of any project materials, participants are thanked for their participation and receive a $50 Gift Card as reimbursement for their involvement and time. Following their completion, participants receive a comprehensive list of relevant perinatal support services and organisations for further reference.

### Trial Status

The project began recruiting in October 2018 and data collection is ongoing.

## Data Analysis

### Sample Size and Power

Assuming 5% missing data at T0, 10% missing data at T2, and 15% missing data at T3 (53), a sample size of 90 powered adequately at 82% (two tailed α = 0.05) to detect a medium effect size (*f* = 0.25), which was observed in a recent CBT for insomnia trial on prenatal insomnia (22).

### Data Storage and Management

All data collected are stored using password protection. Participants' personal details are stored separately from responses to questionnaires and telephone interviews and are only accessible to primary investigators and research assistants. Only primary investigators will have access to questionnaire responses and the final dataset. All identifiable information is kept for 7 years following arising publications and will be destroyed after this point.

To promote data quality, data will be screened for accuracy and further enquiries are made and rectified. Participants are encouraged to request further clarification of questionnaire items if unsure and are provided with assistance via email or telephone by the lead researcher (SV). Results will be analysed upon completion of the trial once all data have been collected. Participants are assigned a unique alphanumeric code which is used to link data across time-points. No data monitoring committee will be employed in the current trial due to limited scale, nor will trial auditing be undertaken.

### Statistical Analysis

All analyses will be conducted on an intention-to-treat basis and will be analysed using R (48) and Mplus (77). Missing data is expected in a longitudinal design and will be addressed using full information maximum likelihood in the structural equation modeling framework (78). Descriptive statistics will be used to characterize baseline demographic characteristics, rates of missing data over time and outcomes over time. Exploratory analyses and graphs will be used to identify univariate or multivariate outliers as well as to evaluate normality assumptions for primary and secondary analyses. Extreme outliers will be winsorised.

Primary and secondary outcomes will be examined using latent growth models. Piecewise models will be used to allow different rates of change: from baseline (T0), through mid-point (T2), to immediate post-intervention (T3) called Slope 1, and from immediate post-intervention (T3) to one-month follow-up (T4) called Slope 2. Loadings for Slope 1 will be 0 for T0 (fixed), freely estimated for T2, 1 for T3 (fixed), and 1 for T4 (fixed). Loadings for Slope 2 will be fixed at 0 for T0, T2, and T3 and fixed at 1 for T4. This model is saturated for the outcome means at each timepoint to ensure that change over time is accurately modeled. The primary trial endpoint for all outcomes is Slope 1, which captures change from baseline to post-intervention. Slope 2 captures maintenance of treatment results over the one-month follow-up and is an exploratory endpoint. The residual variance will be constrained to be equal across time (homogenous) and independent (i.e., an independent, homogenous residual structure). Intercepts of manifest variables for the outcomes will be constrained to 0 to allow estimation of the latent random intercept mean. The intercept and Slope 1 variances and covariances will be freely estimated. The variance of Slope 2 and its covariance with the intercept and Slope 1 will be freely estimated if possible, but given it comprises only two timepoints, if there are convergence or estimation problems, the variance of Slope 2 will be constrained to 0. The two stratification factors, ISI (≤ 14 and >14) and infant age (<8 and ≥8 months) will form four grouping conditions: Early Postpartum Low ISI, Late Postpartum Low ISI, Early Postpartum High ISI, and Late Postpartum High ISI. Dummy codes will be created for each, with Early Postpartum Low ISI as the reference group. These dummy codes will be included as covariates to adjust for effect on the random intercept (79, 80).

Two “treatment” variables (one on the presence/absence of CBT, the other presence/absence of LDT) will be included in the models to predict the intercept, and slopes of each piece, with treatment factors constrained to 0 for the intercept to implement “constrained longitudinal data analysis,” which provides a more accurate estimate of treatment effects from RCT with repeated measures (81, 82). This tests whether the change in outcomes over time (from baseline through mid-point to post-intervention) is different with the presence/absence of CBT or LDT. Effect sizes of the group difference at each time point also will be calculated as adjusted, standardized mean differences by taking the model estimated difference between groups at each time point and standardizing by the combined residual and random intercept variance. A robust maximum likelihood estimator, which is robust to non-normal data (83), will be used to derive confidence intervals and statistical inference. Given this and to maintain interpretability of results, outcomes will be transformed only if visual inspection indicates significant departures from normality.

Sensitivity analyses will be conducted replicating the primary analyses but including receipt of sleep interventions (e.g., starting sleep medication/therapy) outside of the study as a covariate to examine whether this alters study results. To explore predictors and mediators of intervention effects (e.g., changes in sleep-related beliefs, social support, treatment adherence), regression, mediation, and moderation analyses will be conducted. The authors may be contacted regarding access to full trial protocol, intervention materials, and statistical code.

### Dissemination

Results are expected to be disseminated through peer-reviewed scientific publications and conferences. If findings are promising, opportunities to further disseminate will be sought out, such as by contacting media (including social media) outlets and healthcare professionals and organisations (e.g., perinatal professionals, maternal, and child health centres) with the aim of being ultimately implemented into perinatal care and accelerating further intervention development. All participants (eligible or ineligible) who had expressed interest receiving trial results will be provided with a summary of findings following trial completion, including any resulting publications that may arise. Researchers who have made significant contributions to the design, conduct, analysis, and reporting of the current trial will be granted authorship for planned and unplanned publications based on trial data.

## Discussion

Despite the high prevalence of postpartum maternal sleep problems (7), existing research of maternal sleep is scarce, especially for the period beyond the initial postpartum months (17). To the best of our knowledge, RCTs of interventions to specifically improve maternal sleep and associated daytime effects beyond the fourth postpartum month have not yet been undertaken. This latter part of postpartum period represents a time when there is a significant drop in the support services available to women. The current trial specifically addresses this gap by targeting first-time mothers from 4 to 12 months postpartum.

Further, this study takes into consideration both the cognitive behavioural and circadian rhythm factors in postpartum sleep, and attempts to address each using interventions shown to be effective in other populations (20, 36). Testing the efficacy of these two different interventions at the same time could help establish evidence base for two different approaches to postpartum sleep disturbance. This could inform the development of future multi-component interventions, as well as interventions that tailor treatment approaches to women's individual characteristics.

Finally, both interventions used in this study are relatively low-cost and highly scalable. If successful, these interventions have the potential to reach large numbers of perinatal women with disturbed sleep in the community and improve their sleep and well-being, representing much-needed avenues of maternal support.

## Ethics Statement

This study involves human participants and was reviewed and ethics approval was obtained by the Monash University Human Research Ethics Committee (9780) and the Department of Education and Training Victoria (2018_003774). The trial was registered prospectively with Australia and New Zealand Clinical Trials Registry (374954). Informed consent was obtained from all participants. The patients/participants provided their written informed consent to participate in this study.

## Author Contributions

SV: conceptualization, methodology, project administration, investigation, writing–original draft, writing–review, and editing. SR: conceptualization, methodology, writing–review, and editing. MD: resources, writing–review, and editing. JW: software, methodology, writing–review, and editing. BB: conceptualization, methodology, resources, software, supervision, writing–review, and editing. Author contributions are compliant with CRediT taxonomy. All authors contributed to the article and approved the submitted version.

## Conflict of Interest

The authors declare that the research was conducted in the absence of any commercial or financial relationships that could be construed as a potential conflict of interest. The reviewer ET declared a shared affiliation with the authors to the handling editor at time of review. The reviewer A-RH declared a shared affiliation with the authors to the handling editor at time of review.

## Abbreviations

CBT: cognitive behavioural therapy
LDT: light dark therapy.
