# Supplementary material for: Cognitive Behavioural Therapy and Light Dark Therapy for Maternal Postpartum Insomnia Symptoms: Protocol of a Parallel-Group Randomised Controlled Efficacy Trial
Source: Front Glob Womens Health. 2021 Jan 15;1:591677. doi: 10.3389/fgwh.2020.591677 (PMC8593939; doi:10.3389/fgwh.2020.591677)
Supplement: Supplementary file 4 [file Data_Sheet_1.DOCX]

Supplementary Material – Screening and Consent

**BRIEF SCREEN**

Thank-you for your interest in the POSSUM Project! Please answer the following questions to see if this project is suitable for you. You will not be identified by this information as responses to the following questions are totally anonymous.

**Please answer the following:**

|  | Yes | No |
| --- | --- | --- |
| Has it been 4 -12 months since you gave birth? |  |  |
| Are you 18 years old or older? |  |  |
| Are you able to communicate in English? |  |  |
| Are you able to receive phone calls? |  |  |
| Do you have access to emails and the internet? |  |  |
| Did you have a singleton pregnancy (no twins, triplets etc.)? |  |  |
| Are you currently pregnant? |  |  |

How many children do you have?

1  2 or more

**If exclusion criteria have not been met, respondents will be navigated to the Explanatory Statement to read over and then the Informed consent form (below)**

**CONSENT FORM**

By ticking the boxes below, I acknowledge that:

- I have read and understood the Information for Participants
- I understand the purposes, procedures and risks of this research project as described in the Participant Information
- I agree to participate in this project according to the conditions in the Participant Information
- I understand that I will be allocated to one of the four (4) project groups, and that as part of the research protocol, I cannot choose which program to enrol in
- I give consent for my phone interviews to be recorded
- I understand that I can contact the researchers if I have any queries about this research
- The researchers have agreed not to reveal my identity and personal details if information about this project is published or presented in any public form
- The information I provide will be kept strictly confidential unless there is a risk or serious danger posed to myself or someone else at which time my information may be disclosed
- I am able to withdraw from the project at any time
- The first questionnaire and phone call will determine my suitability to take part in this study and in the case that I am ineligible, I will be referred onwards appropriately
- Participation in this study is voluntary: I do not have to take part if I do not want to. If you do agree to participate, please fill in the Consent Form below. You will also receive a copy of the Information for Participants and Consent Form via email as a record.

|  | Yes | No |
| --- | --- | --- |
| I agree to participate in this research project |  |  |
| I would like to receive a summary of the project findings at the conclusion of the study |  |  |
| I agree to being contacted for future studies on sleep and postnatal wellbeing |  |  |

**Please enter your contact information below so we can get in touch with you.**

First name: __________

Last name: __________

Email address: __________

Mobile number: __________

Alternative phone number: __________

Please provide your postal address (this will be used to send you your $50 Gift Card upon project completion)

Unit no.: __________

Street/house no.:__________

Street name: __________

Suburb: __________

State: __________

Post Code: __________

**We can contact you over the phone between 8am and 8pm all days of the week.**

If you have preference which day of the week or time of the day to reach you on the phone, please tell us below.  For example, "please do not call me after 6pm", "best time to reach me is weekday mornings". *Leave blank if you do not have any preference.*

__________________________________________________________________________________________________________________________________________________________

**Do you use a voicemail?**

Yes, and please leave a message when I'm not available

No, please do not leave a voicemail on my phone

**After providing these details, respondents will receive the following message:**

**What's next?**

Thank-you again for showing an interest in the POSSUM Project. Our researchers will send you a link shortly with your own personalised link where you are able to complete the first questionnaire to see whether you may be able to participate in the program. 

Please press the NEXT PAGE button below to record your response.

Stay tuned!

The POSSUM team

**If respondents select NO to either 1, 2-6 (of Brief Screen), respond YES to 8 or disclose 2 or more children, they meet criteria for exclusion. They will not be able to continue to the Explanatory Statement/Informed consent, and will receive the following message:**

Based on the responses you have provided, we believe that The POSSUM Project will not be suitable for you at this time. You may click [here](https://mailchi.mp/e14/support-services2) for a list of support services which you may find helpful.

In addition, you may want to visit some of the following sources for further support:

Your GP or local Maternal and Child Health Centre

- [Perinatal Anxiety & Depression Australia (PANDA)](https://www.panda.org.au/)
- [Centre of Perinatal Excellence (COPE)](http://cope.org.au/)
- [beyondblue](https://www.beyondblue.org.au/)
- [Raising Children's Network](https://raisingchildren.net.au)
- [Lifeline (13 11 14)](https://www.lifeline.org.au/)

Please note that if you believe you have responded incorrectly to the questions, please refresh your browser to enter your responses again.

Please press the **NEXT PAGE** button below to record your response.

If you would like to receive more information about this, you may contact us on psych-possum@monash.edu

**If participants select NO to question 1 (of Brief Screen) but no other exclusion criteria have been met, respondents will be asked if their baby is less than 4 months old. If so, they may still provide informed consent and will be contacted with the link to the first questionnaire once their baby is 4 months of age.**

*If it's been less than 4 months since you gave birth, you can still register your interest and we will contact you once you hit the 4-month mark.

Did you give birth less than 4 months ago?*

**If their baby is older than 12 months, they will receive the ineligible message as above.**
