# Supplementary figures and images for: Cognitive Behavioural Therapy and Light Dark Therapy for Maternal Postpartum Insomnia Symptoms: Protocol of a Parallel-Group Randomised Controlled Efficacy Trial
Source: Front Glob Womens Health. 2021 Jan 15;1:591677. doi: 10.3389/fgwh.2020.591677 (PMC8593939; doi:10.3389/fgwh.2020.591677)

Supplementary Material - Sample of CBT Intervention Emails


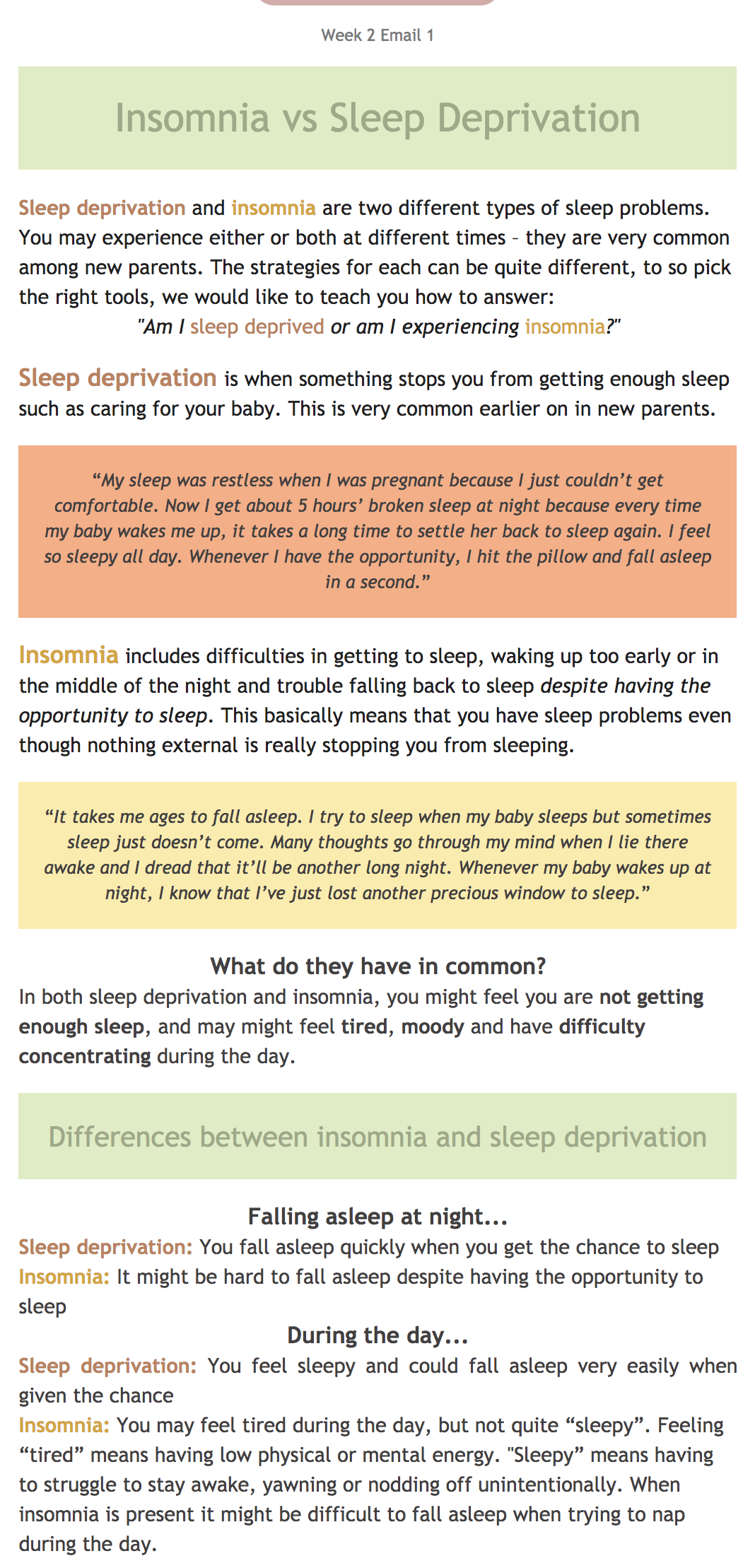

Supplement: Supplementary file 5 [file Data_Sheet_2.DOCX]
